# Psychosocial Suicide Prevention Interventions in the Elderly: A Mini-Review of the Literature

**DOI:** 10.3389/fpsyg.2018.02713

**Published:** 2019-01-09

**Authors:** Patrizia Zeppegno, Eleonora Gattoni, Martina Mastrangelo, Carla Gramaglia, Marco Sarchiapone

**Affiliations:** ^1^Department of Translational Medicine, Università del Piemonte Orientale, Novara, Italy; ^2^Institute of Psychiatry, University Hospital Maggiore della Carità, Novara, Italy; ^3^Department of Medicine and Health Sciences, University of Molise, Campobasso, Italy; ^4^National Institute for Health, Migration and Poverty, Rome, Italy

**Keywords:** suicide, attempted suicide, elderly, psychosocial intervention, mini-review

## Abstract

In Europe the elderly population is projected to increase from 18.5% (93.9 million) in 2014 to 28.7% (149.1 million) by 2080. In the United States it is estimated that by 2030 more than 20% of the population will be aged 65 years or over. This specific population is at high risk of unrecognized or untreated psychiatric illnesses and suicide. It is well known that completed suicide rate increases with age in both men and women. Although elderly people attempt suicide less often than other age groups, they show a higher completion rate. Generally, the methods chosen by elderly are more lethal, the intent is more serious, they are more determined, and they show fewer warning signs than the younger population. A recent systematic review and meta-analysis of psychosocial intervention, following self-harm in adults, found that cognitive behavioral therapy was the most effective therapy in these patients. Unfortunately, there have been few reported trials of other potentially effective interventions. Because the scientific literature on psychosocial suicide prevention interventions in the elderly is still scant, we conducted a mini-review in order to take stock of the situation. Studies were identified through electronic searches of the Cochrane library, MEDLINE, Scopus and the Web of Science databases. PRISMA guidelines were followed and only seven articles met the inclusion criteria. No firm conclusions can be drawn about this topic because there is still very little data and studies use inconsistent outcome measures and designs. Nonetheless, the existing data suggests that psychosocial interventions are promising.

## Introduction

According to the World Health Organisation’s (WHO) estimates, 800,000 people die from suicide every year (WHO, 2017). Although in some countries the suicide rate is higher amongst the young (15–29 years old), generally people aged 70 years and over have the highest suicide rate. When it comes to gender differences, men over 50 years old are particularly at risk and women 70 years old and over are twice as likely to die through suicide than younger (15–29 years old) women (Pearson and Conwell, 1995; WHO, 2014).

In Europe the elderly population is projected to increase from 18.5% (93.9 million) in 2014 to 28.7% (149.1 million) of the population by 2080 [Eurostat (n.d.)] and it is estimated that by 2030 more than 20% of the United States population will be aged 65 years or over. The elderly population is at a high risk of unrecognized or untreated psychiatric illnesses and suicide. In 2012 there was about one elderly suicide every 80 min in the United States (Ortman et al., 2014).

It is widely acknowledged that compared to other age groups, attempted suicide is less common in the elderly, whereas completed suicide is more common, probably due to the choice of more lethal means (McIntosh, 1994). Moreover, it is noticeable that intention to die tends to be greater in elderly suicide cases and elderly people usually show fewer warning signs than the younger population (Bennett and Collins, 2001). Risk factors underlying elderly suicidal behavior include: psychiatric illness (particularly affective disorders), recent loss, alcohol abuse, social isolation, perceived meaning of physical illness (pain, impact on global function), family discord, retirement, cognitive deficits, and institutionalization (Linden and Barnow, 1997; Conwell et al., 2002; Duberstein et al., 2004; Dombrovski et al., 2008).

Given this pattern of risk factors, prevention and intervention strategies targeting the specific needs of the elderly population are recommended. The current global trend in aging means it is necessary to deal with the peculiarities and specific needs of the elderly population and to plan *ad hoc* structured interventions aimed at reducing their suicide risk. Age-sensitive interventions (including psychiatric treatment if necessary), focused on physical health perception and facilitating adjustment to change, may be helpful in reducing elderly suicide risk (Crandall et al., 2007). Psychosocial interventions could be useful, including traditional psychotherapy interventions, as well as self-help groups, case management strategies and psychosocial rehabilitation techniques.

A recent systematic review and meta-analysis of psychosocial interventions following self-harm in adults, suggested that cognitive behavioral therapy is the most effective approach in these patients (Hawton et al., 2016). Unfortunately, there is a dearth of literature on psychosocial interventions focused on suicide prevention in the elderly.

Our aim was therefore to perform a mini-review of the literature on suicide prevention in the elderly, using the search string shown in Appendix 1.

## Materials and Methods

### Selection of Studies

The inclusion criterions were:

• Description or report of psychosocial intervention(s) with a focus on preventing suicidal thoughts and behaviors in elderly populations.
• Design: randomized controlled trial (RCTs), quasi-experimental (e.g., non-randomized controlled studies and before-and-after studies), observational, case-report.

Exclusion criteria were:

• Reviews and meta-analyses.
• Papers not written in English.

### Data Sources and Search Strategy

Four electronic databases (PubMed, Scopus, Cochrane Library, and the Web of Science) were searched from their inception up to 31st January 2018, with the search strings reported in Appendix 1. Articles were selected in accordance with the Preferred Reporting Items for Systematic Reviews and Meta-Analyses (PRISMA) guidelines (Moher et al., 2015; Shamseer et al., 2015).

Two reviewers (EG and MM) independently selected titles, abstracts and full-text publications using the inclusion and exclusion criteria specified above. Disagreements were resolved through discussion with a third reviewer (CG).

We extracted the following information from all publications: country, design, characteristics of study participants, characteristics of the psychosocial interventions described, and a summary of the main study findings which focused on suicide. Ethical statements were not provided in the studies included.

## Results

**Figure 1:**
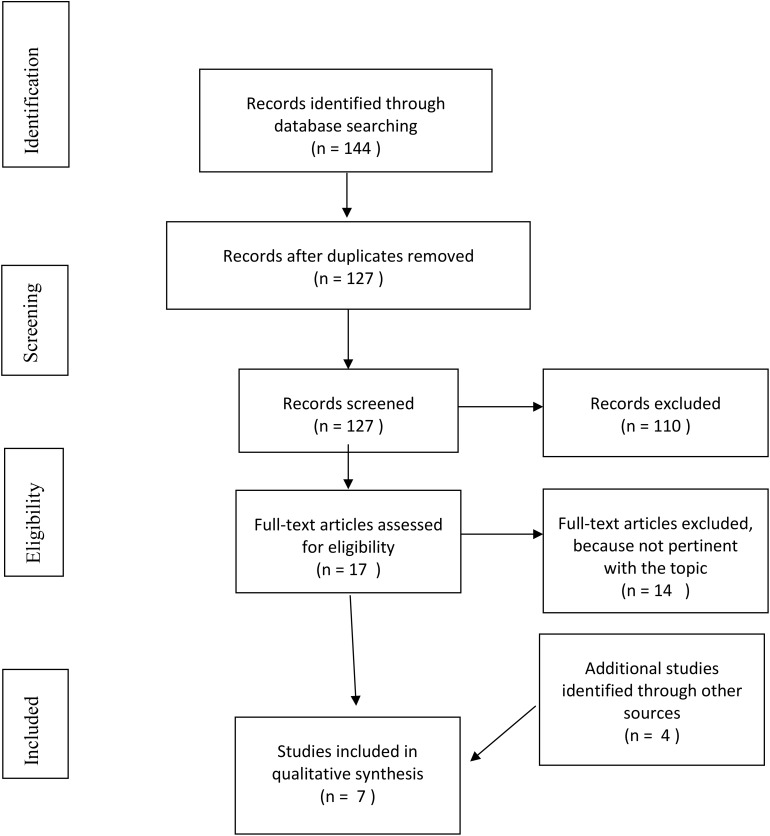
PRISMA flowchart.

The search retrieved 144 articles; 127 articles remained after duplicates were removed. Twelve articles were excluded because they were not written in English, 23 were excluded based on the title (not relevant to the topic) and six were excluded due to the type of publication (reviews and meta-analyses). A further 68 articles were excluded based on the abstract (not relevant) and one full text article was not available. Most of the articles which were excluded were not focused on the selected study population (elderly population), on the selected intervention (psychosocial intervention) or on the intervention target (suicide prevention). Three eligible articles were identified through the database search and four from other sources, thus seven articles were included in this mini-review. The PRISMA flowchart is shown in Figure 1.

One of the selected articles (Bruce and Pearson, 1999) described the Prevention of Suicide in Primary Care Elderly: Collaborative Trial (PROSPECT), a study involving 18 primary care practices that aimed to promote the recognition and treatment of depression and identifying depression as one of the most important risk factors for suicide behaviors in the elderly population. PROSPECT implemented procedures designed to facilitate the use of an algorithm for detecting and treating depression. The PROSPECT algorithm recommended antidepressant pharmacotherapy as the first-line intervention and psychotherapy – in particular interpersonal therapy – as the second-line treatment. At each step the physician could request a psychiatric consultation or refer the patient to a specialist. The PROSPECT intervention added a mental health specialist to the primary care setting. The role of these specialists was to obtain information from patients that would help physicians recognize depression, to offer recommendations and to help educate patients, families and physicians about depression and suicidal ideation. The patients involved were >60 years old, cognitively intact and not institutionalized. Telephone screening for depression was carried out using the Center for Epidemiologic Studies Depression (CESD) scale. People identified with having depression and a small percentage of patients without depression were recruited and invited to a face-to-face interview, to assess depression and other clinical, neuropsychological and social variables necessary for the evaluation of depression and suicide risk. After the assessment the patient’s chart was reviewed by a mental health specialist, who then contacted the physician with recommendations after which intervention procedures started. Recruited patients were contacted again by telephone 4 and 8 months later, in order to assess depressive symptoms, suicide ideation and use of health care services.

In a later article, Bruce et al. (2004) compared the method described above with usual care. Patients who received the PROSPECT intervention showed a greater decrease in depressive symptoms than those who received the usual care (*p* < 0.001). Moreover, a faster reduction in suicidal ideation rate was observed in the PROSPECT group (*p* = 0.01).

Another article (Fiske and Arbore, 2001) evaluated a geriatric outreach program for older people potentially at risk of suicide. The intervention group comprised of 148 elderly people (*M* = 76.9 years, *SD* = 9.0) and the control group of 70 individuals (mean age ± SD, 73.6 ± 7.9). The program consisted of scheduled calls that included well-being checks and emotional support, and weekly home counseling visits. Participants also had access to a 24 h telephone hotline. Suicide risk was evaluated by asking participants about their current and prior suicidal behavior (e.g., “Have you ever thought of killing yourself?”, “Have you ever tried to kill yourself?”, “Are you feeling suicidal now?”), 42% of the intervention group had a lifetime history of suicidal thoughts and 14% had attempted suicide previously; in the control group the corresponding percentages were 30 and 7%. Participants who reported a history of suicidal thoughts were more likely to have a high level of depressive symptoms (measured with the short form of the Geriatric Depression Scale; *p* < 0.01) and a high level of hopelessness (measured with the Geriatric Hopelessness Scale; *p* < 0.01). The 218 participants were contacted for another interview after 1 year. The results showed that after the intervention, the intervention group showed a reduction in hopelessness levels, which may indicate a reduction in suicide risk given that numerous studies have shown a link between hopelessness and suicidality.

Heisel et al. (2009, 2015)conducted a pre- to post-treatment psychotherapy trial involving 17 older patients (*M* age = 70.1 years, *SD* = 5.4) who were assessed for multiple late-life suicide risk factors using specific tests. Suicidal ideation was evaluated with the Geriatric Scale for Suicide Ideation (GSIS). Participants were offered a course of 16 weekly 50 to 60-min sessions of outpatient interpersonal psychotherapy, tailored to older adults at risk for suicide. Participants were helped to identify the factors that were contributing to their psychological pain and to alleviate feelings of desperation, which are common amongst depressed older patients, and opportunities to enhance social connections were identified. In the later phase of treatment, patients were encouraged to continue to develop interpersonal relationships and meaningful post-therapy pursuits. Participants, when they wished, were allowed to continue with therapy after the 16 scheduled sessions. A safety protocol was implemented to try to prevent potentially dangerous situations; this provided suggestions for family members or caregivers of the participants and 24/7 mobile phone access to the study therapist in case of emergency. The intervention produced reductions in suicide ideation (*p* = 0.003) and severity of depression symptoms (*p* = 0.001) whilst social adjustment, enjoyment of social and leisure activities increased (*p* = 0.001).

Kiosses et al. (2015) compared the efficacy of Problem Adaptation Therapy (PATH) and Supportive Therapy for Cognitively Impaired Older Adults (ST-CI) in a randomized controlled trial involving 39 older adults (age ≥65 years; *M* = 82.5, *SD* = 7.16) with unipolar, non-psychotic major depressive disorder, mild or moderate dementia and disability. Both psychosocial interventions were home-delivered and lasted 12 weeks. PATH is designed to reduce depression, depression-related symptoms and disability in elderly patients with depression and cognitive impairment, using a problem-solving approach, compensatory strategies, tools (e.g., calendar, notes, signs, notebook) and environmental adaptations. If the patient was unable to improve his or her emotional regulation with the help of the PATH tools, then the caregiver was asked to get involved. ST-CI is a non-specific therapeutic approach including empathetic listening, encouragement and understanding. It is based on Carl Rogers’ theory and differs from psychotherapies such as problem-solving therapy, interpersonal therapy, or cognitive behavioral therapy. Both the approaches were originally developed for patients with cognitive impairment. Participants’ suicidal ideation was assessed at baseline and every 4 weeks thereafter, using the suicide item (#16) of the Cornell Scale for Depression in Dementia (CSDD). After 12 weeks of treatment both the PATH and ST-CI groups showed similar reductions in suicidal ideation (*p* = 0.002).

The final study (Sirey et al., 2016) compared a service referral control condition, with an Open Door intervention in 161 older adults (age ≥60 years), who were eligible for home meal service in the United States. In contrast with the previous study, cognitive impairment (Mini Mental State Exam <24) was an exclusion criterion. Patients were randomized to the service referral group (*M* = 81 years, *SD* = 9.5) or the Open Door group (*M* = 82.9 years, *SD* = 9.0). The Open Door intervention is a brief, five-step, individualized psychosocial intervention designed to improve depression and its correlates. Sessions are delivered by a counselor who uses motivational interviewing to activate the individual’s wish to seek help. Both interventions lasted 8 weeks (three face-to-face visits during the first 6 weeks and one telephone meeting after 15 days) and suicidal ideation was evaluated using the Suicide Risk Assessment scale. Almost one third (26.7%) of all participants reported suicidal ideation at the baseline. No significant association was found between suicide risk and ethnicity, age, gender, or education. Unfortunately, suicide risk was not evaluated after the intervention.

## Discussion

Some of the psychosocial interventions investigated in the selected articles appear to be effective in reducing suicidal ideation in the elderly, namely the PROSPECT intervention, PATH, ST-CI and outpatient interpersonal psychotherapy tailored to older adults (Bruce and Pearson, 1999; Bruce et al., 2004; Heisel et al., 2009, 2015; Kiosses et al., 2015). One study did not measure the impact of the psychosocial intervention on suicide risk, but argued that the observed reduction in hopelessness levels should translate into lower suicide risk (Fiske and Arbore, 2001). Finally, in one of the studies, suicide risk was assessed before the intervention, when about one third of participants reported suicidal ideation, but not after the intervention (Sirey et al., 2016).

Given the high risk of suicide in the elderly (WHO, 2014), the paucity of literature on psychosocial interventions designed to prevent suicide in the elderly is surprising. This mini-review found only seven relevant studies, some rather outdated and others more recent. The studies were diverse with respect to intervention and assessment methods. The PROSPECT intervention investigated the impact of physician knowledge in the primary care setting (pharmacotherapy and/or interpersonal therapy) and depression care managers (Bruce and Pearson, 1999; Bruce et al., 2004). Another study used a telephone-based geriatric outreach program to provide emotional support to older people considered at risk for suicide (Fiske and Arbore, 2001). The PATH intervention relied instead on a problem-solving approach and ST-CI was based on the Carl Rogers’ theory. The Open Door intervention Heisel et al. (2009, 2015) used interpersonal psychotherapy tailored to older adults at risk for suicide, to help participants deal with psychological pain and desperation. The samples of older adults recruited in the studies were also very diverse; Fiske and Arbore recruited a slightly younger sample (*M* = 76.9 years, *SD* = 9.0) than the others. Some of the interventions described were tailored specifically to older adults with dementia (Kiosses et al., 2015), whereas other studies specifically excluded patients with cognitive impairment (Sirey et al., 2016). This heterogeneity means that the interventions cannot be compared.

Given that suicide risk is high in the elderly population and we live in an aging society, suicide prevention in the elderly is a topic that deserves more attention. It is notable that in the elderly suicide and the wish to die may be related to anxiety, social isolation, loneliness, pain, disability and institutionalization as well as to specific psychopathological conditions, such as depression, which can be treated psychopharmacologically (Linden and Barnow, 1997; Almeida et al., 2012). The former factors might be more amenable to psychosocial treatments than psychopharmacology. Psychosocial interventions should be encouraged given the limitations of medical treatment in older people, for example the moderate effects, drug-drug interactions and increased vulnerability to side effects (Dines et al., 2014).

Although there have been several studies of psychosocial interventions in adults (Hawton et al., 2016), research focusing specifically on elderly people is scarce. The few studies we found, their limitations notwithstanding, suggest that psychosocial interventions have promise as a method of reducing suicide in the elderly. In conclusion, suicide risk assessment should be part of the standard psychiatric assessment in geriatric patients and it is important that effective psychosocial suicide prevention interventions are developed to target the specific needs of this population (Aftab and Shah, 2017).

We found few studies, and the heterogeneity of samples and assessment methods made it difficult to compare the interventions described, but overall the results were promising. Articles reviewed suggested that it is important to assess suicide risk in older people. Once people at risk are identified, appropriate psychosocial interventions should be adopted. Furthermore, there is a need for studies which implement appropriate follow-up, including re-evaluation of suicide risk after intervention. Finally, as the two most important parameters in the field of psychosocial rehabilitation, efficacy, and cost-effectiveness should be taken into account in studies dealing with this topic.

## Author Contributions

All authors contributed to the conception and design of the study, manuscript revision, read, and approved the submitted version. EG and MM reviewed the articles and wrote the first draft of the manuscript.

## Conflict of Interest Statement

The authors declare that the research was conducted in the absence of any commercial or financial relationships that could be construed as a potential conflict of interest.

## Appendix 1

**Table:**

Search engine	Search string
Cochrane library	*(“suicide”) AND (“psychosocial intervention”) AND (“elderly”)*
PubMed	*[(suicide) AND psychosocial intervention] AND elderly*
Scopus	*[TITLE-ABS-KEY (suicide) AND TITLE-ABS-KEY (psychosocial AND intervention) AND TITLE-ABS-KEY (elderly)]*
Web of science	*“suicide” AND “psychosocial intervention” AND “elderly”*

## References

[B1] Aftab A. Shah A. A. (2017). Behavioral emergencies: special considerations in the geriatric psychiatric patient. *Psychiatr. Clin. North Am.* 40 449–462. 10.1016/j.psc.2017.05.010 28800801

[B2] Almeida O. P. Draper B. Snowdon J. Lautenschlager N. T. Pirkis J. Byrne G. (2012). Factors associated with suicidal thoughts in a large community study of older adults. *Br. J. Psychiatry* 201 466–472. 10.1192/bjp.bp.112.110130 23209090

[B3] Bennett A. T. Collins K. (2001). Elderly suicide: a 10-year retrospective study. *Am. J. Forensic Med. Pathol.* 22 169–172. 10.1097/00000433-200106000-00011 11394752

[B4] Bruce M. L. Pearson J. L. (1999). Designing an intervention to prevent suicide: PROSPECT (prevention of suicide in primary care elderly: collaborative trial). *Dialogues Clin. Neurosci.* 1 100–112. 22033641 10.31887/DCNS.1999.1.2/mbruce PMC3181574

[B5] Bruce M. L. Ten Have T. R. Reynolds C. F. III Katz I. I. Schulberg H. C. Mulsant B. H. (2004). Reducing suicidal ideation and depressive symptoms in depressed older primary care patients: a randomized controlled trial. *JAMA* 291 1081–1091. 10.1001/jama.291.9.1081 14996777

[B6] Conwell Y. Duberstein P. R. Caine E. D. (2002). Risk factors for suicide in later life. *Biol. Psychiatry* 52 193–204. 10.1016/s0006-3223(02)01347-1 12182926

[B7] Crandall M. Luchette F. Esposito T. J. West M. Shapiro M. Bulger E. (2007). Attempted suicide and the elderly trauma patient: risk factors and outcomes. *J. Trauma* 62 1021–1027; discussion 1027–1028. 10.1097/01.ta.0000229784.88927.6e 17426562

[B8] Dines P. Hu W. Sajatovic M. (2014). Depression in later-life: an overview of assessment and management. *Psychiatr. Danub.* 26 78–84. 25413518

[B9] Dombrovski A. Y. Butters M. A. Reynolds C. F. III Houck P. R. Clark L. Mazumdar S. (2008). Cognitive performance in suicidal depressed elderly: preliminary report. *Am. J. Geriatr. Psychiatry* 16 109–115. 10.1097/JGP.0b013e3180f6338d 18239196 PMC2671399

[B10] Duberstein P. R. Conwell Y. Conner K. R. Eberly S. Caine E. D. (2004). Suicide at 50 years of age and older: perceived physical illness, family discord and financial strain. *Psychol. Med.* 34 137–146. 10.1017/S0033291703008584 14971634

[B11] Eurostat (n.d.). *People in the EU – Population Projections - Statistics Explained.* Available at: https://ec.europa.eu/eurostat/statistics-explained/index.php/People_in_the_EU_-_population_projections

[B12] Fiske A. Arbore P. (2001). Future directions in late life suicide prevention. *OMEGA* 42 37–53. 10.2190/3t4g-t5u2-q724-e0k8 28938218

[B13] Hawton K. Witt K. G. Taylor Salisbury T. L. Arensman E. Gunnell D. Hazell P. (2016). Psychosocial interventions for self-harm in adults. *Cochrane Database Syst. Rev.* 5:CD012189. 10.1002/14651858.CD012189 27168519 PMC8786273

[B14] Heisel M. J. Duberstein P. R. Talbot N. L. King D. A. Tu X. M. (2009). Adapting interpersonal psychotherapy for older adults at risk for suicide: preliminary findings. *Prof. Psychol. Res. Pract.* 40 156–164. 10.1037/a0014731 20574546 PMC2889497

[B15] Heisel M. J. Talbot N. L. King D. A. Tu X. M. Duberstein P. R. (2015). Adapting interpersonal psychotherapy for older adults at risk for suicide. *Am. J. Geriatr. Psychiatry* 23 87–98. 10.1016/j.jagp.2014.03.010 24840611 PMC4211998

[B16] Kiosses D. N. Rosenberg P. B. McGovern A. Fonzetti P. Zaydens H. Alexopoulos G. S. (2015). Depression and suicidal ideation during two psychosocial treatments in older adults with major depression and dementia. *J. Alzheimers Dis.* 48 453–462. 10.3233/JAD-150200 26402009 PMC5822689

[B17] Linden M. Barnow S. (1997). The wish to die in very old persons near the end of life: a psychiatric problem? Results from the berlin aging study. *Int. Psychogeriatr.* 9 291–307. 10.1017/s1041610297004456 9513029

[B18] McIntosh J. L. (1994). *Elder Suicide: Research, Theory, and Treatment.* Washington, DC: American Pychological Association 10.1037/10159-000

[B19] Moher D. Shamseer L. Clarke M. Ghersi D. Liberati A. Petticrew M. (2015). Preferred reporting items for systematic review and meta-analysis protocols (PRISMA-P) 2015 statement. *Syst. Rev.* 4:1. 10.1186/2046-4053-4-1 25554246 PMC4320440

[B20] Ortman J. M. Velkoff V. A. Hogan H. (2014). *An Aging Nation: The Older Population in the United States.* Current Population Reports No. P25-1140 Washington, DC: United States Census Bureau.

[B21] Pearson J. L. Conwell Y. (1995). Suicide in late life: challenges and opportunities for research. Introduction. *Int. Psychogeriatr.* 7 131–136. 10.1017/S104161029500192X 8829422

[B22] Shamseer L. Moher D. Clarke M. Ghersi D. Liberati A. Petticrew M. (2015). Preferred reporting items for systematic review and meta-analysis protocols (PRISMA-P) 2015: elaboration and explanation. *Br. Med. J.* 350:g7647. 10.1136/bmj.g7647 25555855

[B23] Sirey J. A. Banerjee S. Marino P. Halkett A. Raeifar E. Paggi M. (2016). Improving mental health treatment initiation among depressed community dwelling older adults. *Am. J. Geriatr. Psychiatry* 24 310–319. 10.1016/j.jagp.2015.11.005 26915900 PMC8178741

[B24] WHO (2014). *First WHO Report on Suicide Prevention.* Available at: http://www.who.int/mediacentre/news/releases/2014/suicide-prevention-report/en/

[B25] WHO (2017). *Suicide Data.* Available at: http://www.who.int/mental_health/prevention/suicide/suicideprevent/en/

